# 

**Published:** 1897-08-28

**Authors:** 


					THE HOSPITAL. ABSOLUTELY PURE, therefore BEST. Acoust 28m, 1897.
CADBURY'S -g* "Of OAR?HFY'S
GOCOA ^11 Jlnll COCOA
? The standard of highest purity at present (2*k NO ALKALIES USED,
attainable."?Lancet.
HdLASOOir WESTERN IHF IBM ART
No. 570; Vol. XXII. Saturday,
August 28th, 1897.
iwUfU.UAJ\.AMB J\Uri niLti ?tU&J-AJAA '
[SubscriptionTerms,] pEiICE TWOPENCE.

				

